# A rare case of extensive cervico-thoracic ossification of the posterior longitudinal ligament causing myelopathy

**DOI:** 10.1051/bmdcn/2018080427

**Published:** 2018-11-26

**Authors:** Arun-Kumar Kaliya-Perumal, Mark Tan, Jacob Yoong Leong Oh

**Affiliations:** 1 Department of Orthopaedic Surgery, Spine Division, Tan Tock Seng Hospital Singapore; 2 Department of Orthopaedic Surgery, Melmaruvathur Adhiparasakthi Institute of Medical Sciences and Research Tamil Nadu India

**Keywords:** Cervical spondylosis, Myelopathy, Neurodegenerative diseases, Ossification of posterior longitudinal ligament, Spine

## Abstract

Ossification of the posterior longitudinal ligament (OPLL) most commonly occurs in the cervical spine, usually involving two to three segments; however, the disease has the potential to occur anywhere in the spine. We encountered a fifty-one year old male with progressive unsteadiness and bilateral lower limb weakness for a period of six months which eventually became worse resulting in inability to walk without assistance. Neurological examination revealed normal upper limb function; however, the lower limbs demonstrated motor dysfunction. Signs of myelopathy were elicited and the patient was subjected to detailed radiological evaluation. CT and MRI scans revealed an extensive cervico-thoracic continuous OPLL from C3 to T3 causing significant cord compression. In view of the deteriorating neurological status, extensive C3-T3 laminectomy with instrumented posterolateral fusion was done and the patient recovered without any immediate or delayed C5 palsy. This case highlights a rare occurrence or extensive OPLL involving eight segments at the cervico-thoracic region. This report also discusses surgical strategies for managing such extensive presentations and our technique to prevent C5 palsy.

## 1. Introduction

Ossification of the posterior longitudinal ligament (OPLL) commonly occurs in the cervical spine and less commonly in the thoracic spine, predominantly affecting the Asian population [1, 2]. However, extensive continuous OPLL involving the cervico- thoracic spine is unusual and is rarely described in the literature. In such cases, when surgical decompression is indicated in view of the deteriorating neurological status, it becomes a challenge for the operating surgeon to perform extensive posterior decompression and provide adequate stabilization, while taking into consideration the high risk of C5 palsy due to the posterior shifting of the spinal cord [3–7]. Here, we report a patient with extensive C3 to T3 OPLL who was managed successfully with posterior decompression and instrumented fusion. The patient subsequently had an uneventful follow up and complete recovery without any immediate or delayed C5 palsy. We intend to report this case considering the extensive nature of the ossification and the successful outcome.

## 2. Case report

A fifty-one years old delivery man with no significant past medical history, presented with progressive unsteadiness and bilateral lower limb weakness over a period of six months which eventually became worse resulting in inability to walk without a walking aid. In addition, he also had chronic neck stiffness for over two years for which he did not seek any intervention. He denied any problem with hand dexterity such as difficulty to use chop sticks, button his shirt or pick up a coin.

A thorough neurological examination was performed which showed significant signs of myelopathy in the lower limbs. In particular, the medical research council’s (MRC) grading of muscle power in both lower limbs (L2-S1) was 4/5. Deep tendon reflexes (DTRs) including the knee jerk and ankle jerk were exaggerated on both lower limbs. Babinski’s sign was positive bilaterally. The patient had difficulty in getting up from an armless chair and was unable to perform a tandem gait. Interestingly, he did not demonstrate any upper limb signs. His sensory-motor function and reflexes were normal in both upper limbs and Hoffman’s sign was negative.

**Fig. 1:**
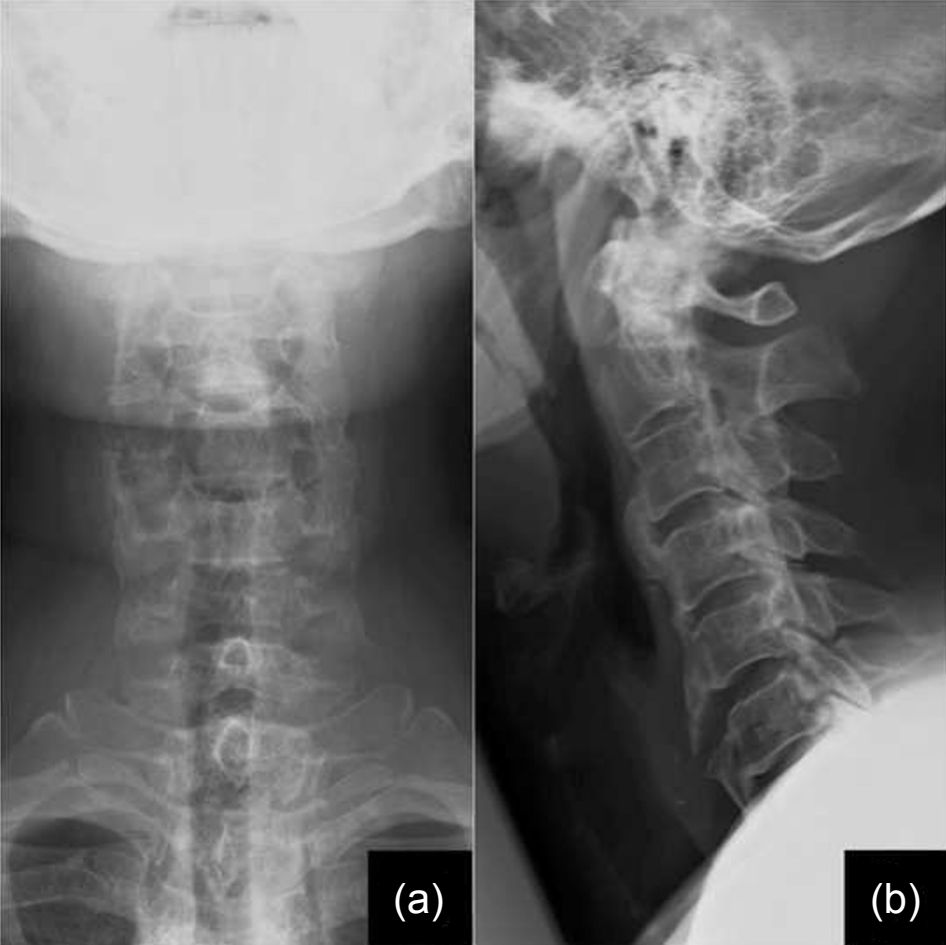
Pre-operative (a) antero-posterior and (b) lateral view X-ray images showing signs of degeneration with loss of cervical lordosis and anterior osteophytes involving C3 to C6.

**Fig. 2:**
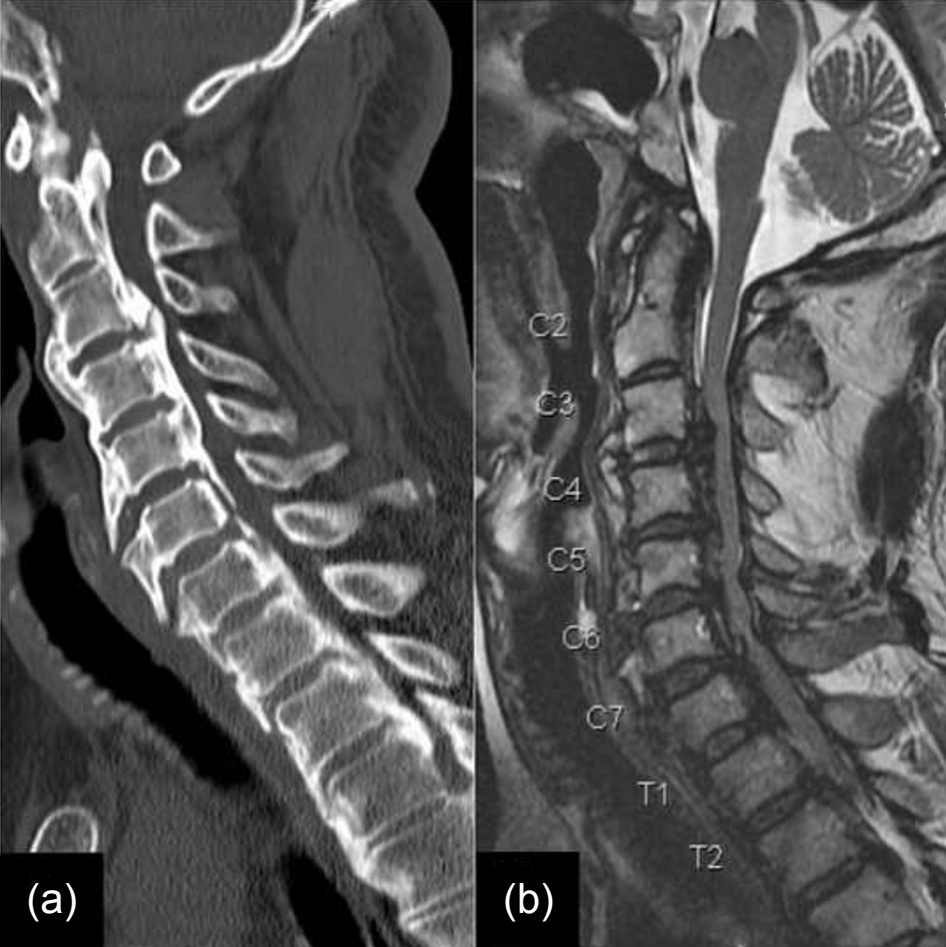
Pre-operative sagittal view (a) CT and (b) MRI images showing ossification of the posterior longitudinal ligament extending from C3 to T3 causing significant cord compression.

**Fig. 3:**
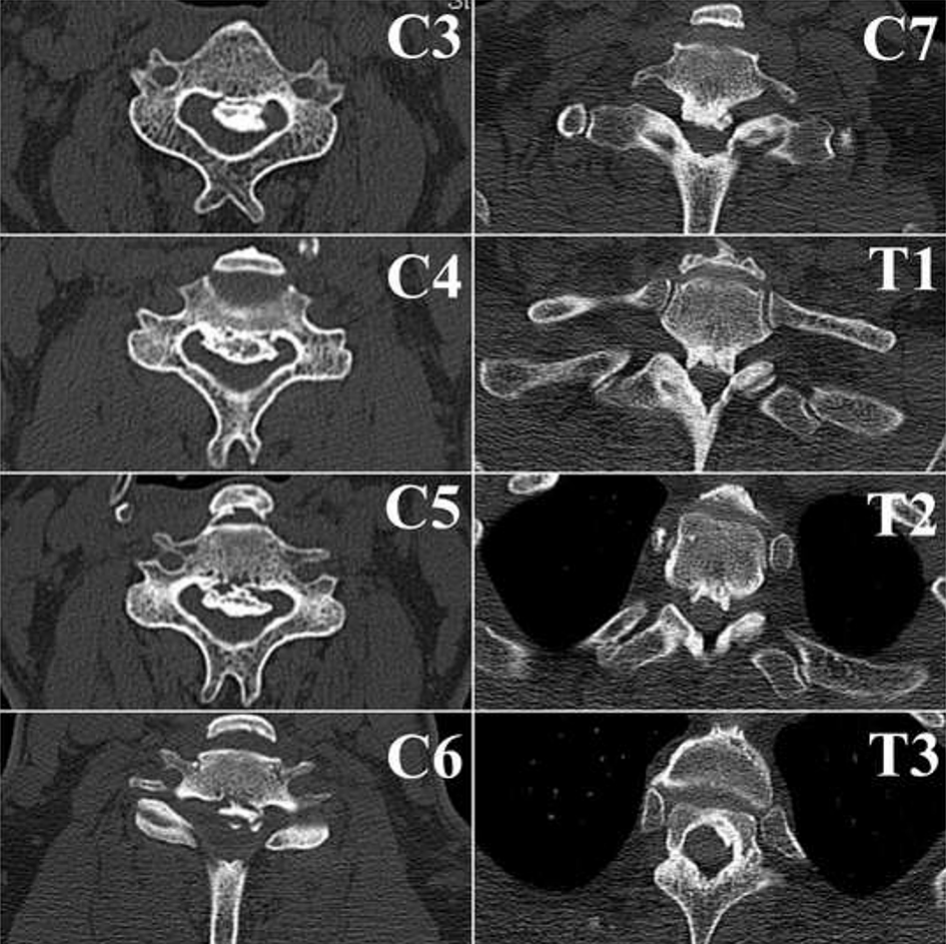
Axial view CT images of all the involved segments showing the amount of canal compromise caused by the OPLL.

The Japanese orthopaedic association (JOA) score was 14/17 indicating grade 1 disability. Xrays of the cervical spine showed signs of degeneration with loss of cervical lordosis and anterior osteophytes involving C3 to C6 (Fig. 1). Computerised tomography (CT) and magnetic resonance imaging (MRI) were suggestive of an extensive OPLL from C3-T3 causing significant canal compromise (Fig. 2 and 3). No cord signal changes were noticeable. A diagnosis of extensive cervico-thoracic OPLL causing myelopathy was made. Considering the clinico-radiological presentation and to prevent any further deterioration of neurological status, immediate surgery was planned.

Dexamethasone 8mg was given intravenously prior to the procedure. A C3-T3 posterior decompression and instrumented postero-lateral fusion using C3-C6 lateral mass screws and T1- T3 pedicle screws was done with O-Arm navigation guidance (Medtronic StealthStation™ S7 surgical navigation system, Medtronic Inc., CO, USA). To reduce the risk of C5 palsy by sudden extensive posterior migration of the cord, laminectomy was performed in three stages with the aid of somatosensory evoked potential (SSEP) and motor evoked potential (MEP) neuro-monitoring. Firstly, the lamina was removed en-bloc from C3-C5 with the aid of a high speed burr. The spinal cord was then allowed to adapt to its new position for 10 minutes before performing similar en-bloc laminectomy for C6-C7. After waiting for another 10 minutes, the final laminectomy for T1-T3 was performed in a piece meal fashion. Postero-lateral fusion was done using local bone chips and bone substitutes. The operative time was 5 hours.

**Fig. 4:**
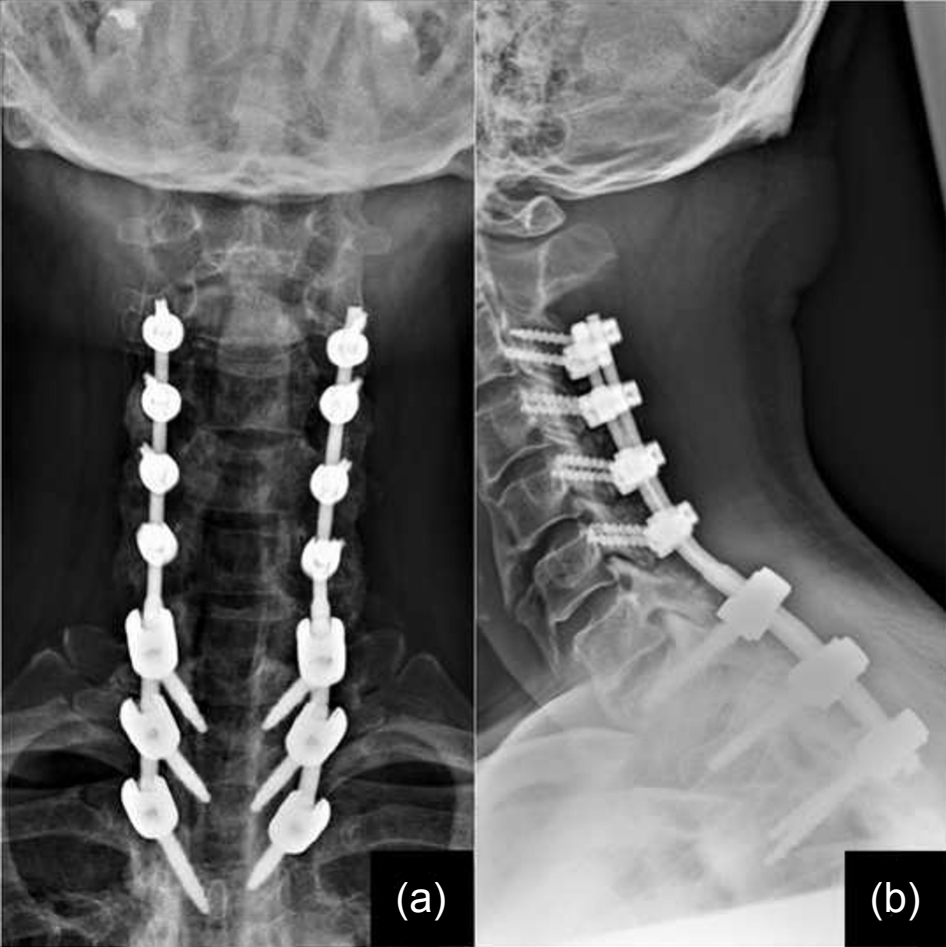
Final follow-up (a) antero-posterior and (b) lateral view X-ray images.

The post-operative period was uneventful and the patient was subjected to physiotherapy as tolerated. He was advised to wear a cervical collar for the first six weeks and was allowed to walk with support from the second post operative day. There was no wound related issues and his upper limb power remained full with no signs of C5 palsy. He was discharged in two weeks. Rehabilitation protocols were continued and his condition gradually improved. At one month follow up, he was able to walk without support and had normal power in both lower limbs. He was followed up every month for the first six months and every 6 months thereafter. He was back to work in 6 months and his X-ray suggested adequate postero-lateral fusion. Throughout his follow-up for 2 years, there was no evidence of implant loosening and his condition remained stable (Fig. 4).

## 3. Discussion

OPLL is a hyperostotic condition characterised by ectopic calcification of the posterior longitudinal ligament [8]. The ligament becomes calcified by enchondral ossification and mature lamellar bone is eventually formed [9]. It is generally classified into four types: continuous, segmental, mixed and localized [10, 11]. It is usually seen at mid and lower cervical regions; however, it can occur anywhere in the spine [12–14]. Most patients present with progressive symptoms of spinal cord compression as in our patient; however, presentations may vary depending upon the site and extent of the ossification [15].

Extensive OPLL spanning over multiple vertebral segments and intervening disc spaces is a rare phenomenon. In such scenarios, the surgical challenge is to adequately decompress the spinal cord without any complications, taking into consideration the various surgical options available. For extensive decompression at the cervico-thoracic region, anterior surgery is least preferred and the appropriate surgical option is either C3-T3 laminoplasty or C3-T3 laminectomy with instrumented postero-lateral fusion. However, multi-level laminoplasty is contraindicated in patients with kyphotic cervical spine as it may lead to further cord compression due to loss of alignment [16, 17].

Therefore, our strategy was to do C3-T3 laminectomy with instrumented postero-lateral fusion [18]. However, when extensive cervical cord decompression by multi-segmental laminectomy is performed, there could be significant posterior shift of the spinal cord leading to transient or prolonged C5 palsy [3–7]. To avoid this, we staged our decompression into three steps: C3-C5, C6- C7 and T1-T3 giving adequate time in between the steps for the cord to adapt to its new position. With neuro-monitoring aid, we were able to look for SSEP and MEP signal changes during each step of the surgery. With this strategy, we believe that the sudden movement of the spinal cord is controlled and occurs gradually, thereby reducing the risk of C5 palsy [19, 20].

## 4. Conclusion

This case report highlights a rare occurrence of extensive cervico- thoracic ossification of the posterior longitudinal ligament leading to worsening myelopathy prompting immediate surgical decompression. Extensive C3-T3 laminectomy with instrumented postero-lateral fusion was done and the patient recovered without any immediate or late complications. This emphasizes the importance of controlled posterior decompression and instrumented fusion for extensive OPLL involving the cervico-thoracic junction.

## Conflict of interest statement

The authors disclose no conflicts of interest.

## References

[1] Abiola R, Rubery P, Mesfin A. Ossification of the Posterior Longitudinal Ligament: Etiology, Diagnosis, and Outcomes of Nonoperative and Operative Management.Global Spine J. 2016; 6(2): 195–204. 26933622 10.1055/s-0035-1556580 PMC4771496

[2] Choi BW, Song KJ, Chang H. Ossification of the posterior longitudinal ligament: a review of literature. Asian Spine J. 2011; 5(4): 267–76. 22164324 10.4184/asj.2011.5.4.267 PMC3230657

[3] Wang T, Wang H, Liu S, Ding WY. Incidence of C5 nerve root palsy after cervical surgery: A meta-analysis for last decade. Medicine (Baltimore). 2017; 96(45): e8560. 29137073 10.1097/MD.0000000000008560 PMC5690766

[4] Kratzig T, Mohme M, Mende KC, Eicker SO, Floeth FW. Impact of the surgical strategy on the incidence of C5 nerve root palsy in decompressive cervical surgery. PLoS One. 2017; 12(11): e0188338. 29145512 10.1371/journal.pone.0188338 PMC5690695

[5] Baba S, Ikuta K, Ikeuchi H, Shiraki M, Komiya N, Kitamura T, et al Risk Factor Analysis for C5 Palsy after Double-Door Laminoplasty for Cervical Spondylotic Myelopathy. Asian Spine J. 2016; 10(2): 298–308. 27114771 10.4184/asj.2016.10.2.298 PMC4843067

[6] Gu Y, Cao P, Gao R, Tian Y, Liang L, Wang C, et al Incidence and risk factors of C5 palsy following posterior cervical decompression: a systematic review. PLoS One. 2014; 9(8): e101933. 25162509 10.1371/journal.pone.0101933 PMC4146468

[7] Liu T, Zou W, Han Y, Wang Y. Correlative study of nerve root palsy and cervical posterior decompression laminectomy and internal fixation. Orthopedics. 2010; 33(8). 10.3928/01477447-20100625-08 20704111

[8] Stapleton CJ, Pham MH, Attenello FJ, Hsieh PC. Ossification of the posterior longitudinal ligament: genetics and pathophysiology. Neurosurg Focus. 2011; 30(3): E6. 10.3171/2010.12.FOCUS10271 21434822

[9] Sato R, Uchida K, Kobayashi S, Yayama T, Kokubo Y, Nakajima H, et al Ossification of the posterior longitudinal ligament of the cervical spine: histopathological findings around the calcification and ossification front. J Neurosurg Spine. 2007; 7(2): 174–83. 17688057 10.3171/SPI-07/08/174

[10] Tanaka M, Kanazawa A, Yonenobu K. Diagnosis of OPLL and OYL: Overview. In: Yonenobu K., Nakamura K., Toyama Y. (eds) OPLL Springer, Tokyo 2006 111–3 p.

[11] Chang H, Kong CG, Won HY, Kim JH, Park JB. Inter- and intraobserver variability of a cervical OPLL classification using reconstructed CT images. Clin Orthop Surg. 2010; 2(1): 8–12. 20190995 10.4055/cios.2010.2.1.8 PMC2824098

[12] Hirai T, Yoshii T, Nagoshi N, Takeuchi K, Mori K, Ushio S, et al Distribution of ossified spinal lesions in patients with severe ossification of the posterior longitudinal ligament and prediction of ossification at each segment based on the cervical OP index classification: a multicenter study (JOSL CT study). BMC Musculoskelet Disord. 2018; 19(1): 107. 29621987 10.1186/s12891-018-2009-7 PMC5887213

[13] Fujimori T, Watabe T, Iwamoto Y, Hamada S, Iwasaki M, Oda T. Prevalence, Concomitance, and Distribution of Ossification of the Spinal Ligaments: Results of Whole Spine CT Scans in 1500 Japanese Patients. Spine (Phila Pa 1976). 2016; 41(21): 1668–76. 27120057 10.1097/BRS.0000000000001643

[14] Li H, Zhou X, Chen G, Li F, Zhu J, Chen Q. Radiological manifestations and surgical outcome of combined upper cervical cord compression and cervical ossification of the posterior longitudinal ligament with a minimum 2-year follow-up. Medicine (Baltimore). 2017; 96(45): e8332. 29137014 10.1097/MD.0000000000008332 PMC5690707

[15] Song KJ, Park CI, Kim DY, Jung YR, Lee KB. Is OPLL-induced canal stenosis a risk factor of cord injury in cervical trauma? Acta Orthop Belg. 2014; 80(4): 567–74. 26280731

[16] Mitsunaga LK, Klineberg EO, Gupta MC. Laminoplasty techniques for the treatment of multilevel cervical stenosis. Adv Orthop. 2012; 2012: 307916. 22496982 10.1155/2012/307916 PMC3310284

[17] Tang HM, Yeh KT, Lee RP, Chen IH, Yu TC, Liu KL, et al Combined expansive open-door laminoplasty with short-segment lateral mass instrumented fusion for multilevel cervical spondylotic myelopathy with short segment instability. Ci Ji Yi Xue Za Zhi. 2016; 28(1): 15–9. 28757711 10.1016/j.tcmj.2015.09.004 PMC5509173

[18] Lin D, Ding Z, Lian K, Hong J, Zhai W. Cervical ossification of the posterior longitudinal ligament: Anterior versus posterior approach. Indian J Orthop. 2012; 46(1): 92–8. 22345814 10.4103/0019-5413.91642 PMC3270613

[19] Jeon HS, Kim KN. Delayed Bilateral C5 Palsy following Circumferential Decompression and Fusion in Patient with Cervical Spon- dylotic Myelopathy. Korean J Spine. 2015; 12(3): 200–3. 26512284 10.14245/kjs.2015.12.3.200 PMC4623184

[20] Lau D, Park P. Delayed C5 Palsy after Laminectomy and Fusion for Ossification of the Posterior Longitudinal Ligament. J Spine. 2012; 1: 104.

